# Supplementary material for: Association between Arousals during Sleep and Subclinical Coronary Atherosclerosis in Patients with Obstructive Sleep Apnea
Source: Brain Sci. 2022 Oct 8;12(10):1362. doi: 10.3390/brainsci12101362 (PMC9599630; doi:10.3390/brainsci12101362)
Supplement: Supplementary file 1 [file brainsci-12-01362-s001.zip › brainsci-1930151-supplementary.pdf]

Supplementary

Table S1 Baseline characteristics of the study population according to the presence of plaque

**Variables**	**Overall (*n*=99)**	**Plaque+ (*n*=62)**	**Plaque- (*n*=37)**	***p*-value**
**Demographic and clinical characteristics**				
Age, years	50.6±9.4	50.8±9.5	50.3±9.5	0.774
Male, n (%)	76 (76.8)	50 (80.6)	26 (70.3)	0.237
Body mass index, kg/m^2	27.7 (25.8, 30.8)	28.7±4.0	27.4±3.7	0.103
Neck circumference, cm	40.9±3.5	41.5±3.5	40.1±3.4	0.062
Smoking status				0.022
Never, n (%)	52 (52.5)	26 (41.9)	26 (70.3)	
Former, n (%)	13 (13.1)	10 (16.1)	3 (8.1)	
Current, n (%)	34 (34.3)	26 (41.9)	8 (21.6)	
Systolic blood pressure, mmHg	133 (125, 147)	136.4±14.4	134.4±17.0	0.519
Diastolic blood pressure, mmHg	83.2±10.6	84.2±10.3	81.5±11.0	0.233
Hypertension, n (%)	56 (56.6)	42 (67.7)	14 (37.8)	0.004
Diabetes mellitus, n (%)	14 (14.1)	10 (16.1)	4 (10.8)	0.463
Hyperlipidemia, n (%)	35 (35.4)	24 (38.7)	11 (29.7)	0.366
Lipid-lowering medication use, n (%)	19 (19.2)	16 (25.8)	3 (8.1)	0.031
Prior history of CAD, n (%)	6 (6.1)	6 (9.7)	0 (0)	0.129
ESS	11 (8, 15)	12 (8, 15)	10 (7, 15)	0.321
**Polysomnography parameters**				
AHI, /h	36.5 (16.5, 61.4)	42.9 (27.5, 65.1)	25.3 (11.0, 46.1)	0.004
ODI, /h	33.4 (16.5, 59.9)	24.4 (8.9, 49.1)	43.4 (21.5, 65.1)	0.015
T90, %	3.5 (0.3, 13.1)	1.5 (0.1, 6.6)	4.7 (0.5, 15.8)	0.065
Lowest SpO2, %	81.0 (72.0, 88.0)	79.0 (68.8, 85.3)	85.0 (74.0, 89.0)	0.07
Mean SpO2, %	94.0 (91.0, 95.0)	93.0 (91.0, 95.0)	94.0 (91.5, 96.0)	0.11
TST, min	409.0 (349.5, 447.5)	409.5 (349.8, 445.6)	408.0 (349.3, 450.3)	0.868
Sleep efficiency, %	78.0 (68.1, 84.4)	78.4 (67.9, 84.6)	77.6 (69.0, 83.7)	0.925
Time in sleep stage, % per TST				
N1	22.9 (16.2, 34.4)	24.9 (18.3, 37.0)	19.1 (11.0, 27.4)	0.01
N2	48.0 (38.7, 52.6)	47.0 (37.2, 51.2)	48.5 (41.0, 56.2)	0.035
N3	13.2 (5.5, 17.3)	13.2 (6.8, 17.1)	13.2 (3.3, 18.6)	0.965
REM	15.6 (11.3, 20.3)	15.4 (10.5, 20.8)	16.0 (12.5, 20.1)	0.487
Arousal index, /h	32.2 (21.3, 47.1)	37.1 (26.1, 49.8)	22.8 (17.8, 38.2)	0.002

Data are presented as n (%), mean ± standard deviation, or median (interquartile range).

AHI, apnea-hypopnea index; CAD, coronary artery disease; ESS, Epworth sleepiness scale; ODI, oxygen desaturation index; REM, rapid eye movement; SpO2, oxygen saturation; TST, total sleep time; T90, percent of night time spent with oxygen saturation below 90%.
